# Tackling climate change and health inequalities in primary care

**DOI:** 10.1093/fampra/cmac146

**Published:** 2022-12-20

**Authors:** David N Blane, Nayanika Basu

**Affiliations:** General Practice and Primary Care, School of Health & Wellbeing, University of Glasgow, Scotland, United Kingdom; Undergraduate Medical School, School of Medicine, Dentistry & Nursing, University of Glasgow, Scotland, United Kingdom

**Keywords:** community medicine, continuing medical education (CME), health disparities, occupational/environmental medicine, primary care

## Abstract

The Climate Emergency is now widely accepted as the biggest public health crisis facing humanity. Previous research has highlighted how social and health inequalities shape the health impacts of climate change in the UK, but there has been little attention to the role of general practice in deprived areas. This Brief Report summarises a roundtable discussion of Scottish ‘Deep End’ GPs - family doctors working in the most socio-economically deprived areas - which took place weeks before the 26th UN Climate Change Conference (COP26), held in Glasgow in November 2021. The report highlights the need for urgent action to make general practice more sustainable, including recommendations for community engagement, (de)prescribing, medical education, digital inclusion, and investment in premises and capacity building.

**Key messages**

- GPs are mindful of the intersections of the Climate Crisis and Health Inequalities.
- Fifteen-minute GP appointments as standard would facilitate shared decision-making.
- Prescribing formularies should take the carbon impact of medications into account.
- Sustainability should feature in undergraduate and postgraduate medical education.

## Introduction

The Climate Emergency is now widely accepted as the biggest public health crisis facing humanity.^1^ Previous research has highlighted how social and health inequalities shape the health impacts of climate change in the United Kingdom,^2^ but there has been little attention to the role of general practice in deprived areas.

Scotland’s Deep End GP practices are practices with the highest percentage of registered patients living in the most socioeconomically deprived postcodes—the majority (roughly 70%) of these practices are based in the city of Glasgow.^3^ Since 2009, the Scottish Deep End Project has been a collaboration between frontline GPs and academics working and advocating to improve the volume and quality of primary care in areas of socioeconomic disadvantage.

In November 2021, world politicians convened in Glasgow for the 26th United Nations Climate Change Conference (COP26). In anticipation, a group of 10 GP colleagues from a variety of Deep End settings contributed to an online roundtable event to explore climate change in the context of health inequalities. Discussion centred on the various factors that influence Deep End patients and practices in particular, but also explored the urgent need for system-wide solutions to tackle this burden. The full report is available on the Deep End website^4^; what follows is a brief summary.

## The role of general practice

The discussion opened with a sense from many participants that GPs are “not experts in climate change” but that there was an urgent need for climate justice and action to future proof and protect the lives of our patients and the services we deliver. Participants reflected that while GPs may lack specific expertise on climate science, in the words of Henry Sigerist, GPs and their teams “*well know the factors that paralyse all their efforts*. *They are not only scientists but also responsible citizens, and if they did not raise their voices, who else should?*”^5^

The group recognized that when our patients are malnourished because of damaging food systems, or when patients struggle with weight, inactivity, and chronic respiratory and cardiovascular conditions because of unhealthy transport systems and environments, or when stressed patients use cigarettes, alcohol, drugs, and violence to cope with economic and social injustices, these are the drivers of climate change intersecting within the communities we serve. Participants expressed the hope that collective action could facilitate shifts to more sustainable approaches and models of care. It was recognized, however, that the most effective drivers of change are upstream and that we need to “*apply pressure at a structural and political level*” to move from “*an unequal, sickness economy*” of unsustainable growth to an economy guided by a compass where every individual enjoys just social foundations, yet humanity collectively operates within viable planetary boundaries.^6^ As we recover from a global pandemic, this shift in thinking will help facilitate a transition to a healthier, fairer, greener, safer future.

## Inclusive change

Despite the urgent need to act, participants recognized that meaningful progress can only be achieved with genuine inclusivity and collectivism, as a radical shift to policies with a climate action focus could attract negative views. Traditional societal markers of “success” have for generations included car ownership, overseas holidays, and numerous gifts for children—advantages that have been disproportionately enjoyed by the most affluent sections of society. One participant raised the point that globally “*the richest 10% produce half of lifestyle carbon emissions, while the poorest 50% produce only a tenth*.”^7^ Shifting cultural norms and expectations will not be easy.

In the health sector, there was agreement that improved knowledge sharing and shared decision tools could help staff and patients to engage in a patient-centred approach for greener, lower carbon health interventions. Primary care networks (GP clusters in Scotland) could build on the potential of community-oriented primary care teams to engage with schools, families, and communities to improve public health at a local level. Collaborative and inclusive decision-making with patients was felt to be pivotal to any successful local neighbourhood and system-wide changes. For individual practices and practitioners, participants proposed standing in solidarity with patients, amplifying their voices on issues that affect them, and ensuring that change is introduced with humility, sensitivity, and a sense of collective action and trust. Practical examples included improved insulation in housing to reduce fuel poverty, improved active travel options, improved access to green spaces, improved access to technology to facilitate access to health care, and other essential services.

## Sustainable general practice

At its core, sustainable general practice is based on preventative medicine. The principles of “realistic medicine,” outlined in the Chief Medical Officer for Scotland’s annual reports since 2016, include reducing health care-generated waste (e.g. carbon contributions), addressing unwarranted variation in practice and outcomes, and building a more personalized approach to care with a focus on shared decision-making.^8^ Participants supported the wider rollout of resources such as the RCGP Greener Impact for Health toolkit,^9^ which align with these principles.

The shift in focus to sustainability and wellbeing at Scottish Government and Local Authority levels was welcomed. It was recognized that both in Scotland^10^ and UK-wide,^11^ there is higher air pollution in more deprived areas, despite lower levels of vehicle ownership. Participants agreed that joined up working with councils and local grassroots groups could improve the safety of pedestrianized and cycling networks surrounding GP practices, encouraging active travel amongst staff and patients. It was also recognized that increased advocacy of active travel, equitable access to green space, and nonpharmacological methods of improving health and wellbeing by primary care teams empowers patients toward better health, while reducing the collective climate footprint. UK appointment length differs from comparable high income health systems.^12^ Participants favoured the trend for 15-min GP appointments as a minimum standard, against the UK norm of 10 min, to enhance opportunities for social prescribing and work with embedded support workers, to prioritize social wellbeing, financial wellbeing, mental health and personalized, holistic care.^13^

## Prescribing and deprescribing

It was recognized that prescribing has the single largest carbon impact in general practice.^14^ As well as promoting nonpharmacological approaches to health and wellbeing, participants agreed on the need for more rational prescribing, and deprescribing where appropriate. In particular, it was noted that inhaler prescriptions alone account for approximately 13% of the carbon footprint of General Practice.^15^ Careful work to switch from carbon-intense Metered Dose Inhalers (MDIs) to Dry Powder Inhaler (DPI) alternatives is already being carried out in practices, but it was agreed that this work requires consistent support from health boards and widespread dissemination of helpful resources.^16,17^

There was an accompanying call for action at formulary level for the carbon impact of medicines to be considered and for sustainable prescribing to be promoted via formularies and toolkits with the carbon cost of medications outlined, sharing examples of good practice.^18^ Similarly, the use of well-designed decision aids^19,20^ could help both patients and clinicians to choose more sustainable medications across a range of disease areas, in line with “realistic medicine” principles.^8^

## Digital inclusion

The use of digital technology and telemonitoring by health care teams provides opportunities to reduce patient and staff travel and associated carbon footprint. However, participants cautioned that decision makers be mindful of unintended widening of health inequalities. Ongoing evaluation of the inequalities impact of remote consulting is needed.^21,22^

## Medical education

Participants agreed that sustainability should be part of teaching and training at undergraduate and postgraduate levels. Climate change and health (and health inequalities) should be integrated in sustainable health care themes across curricula. Participants were supportive of initiatives such as the “Planetary Health Report Card,”^23^ which compares medical school teaching of planetary health, with recommendations for change at the institutional level. Other opportunities for Quality Improvement (QI) projects with a sustainability focus should be encouraged at both undergraduate (e.g. during “Student Selected Components” [SSCs]) and postgraduate levels, with improved mechanisms for sharing QI project ideas across practices and clusters/networks. Supported education time and posts for sustainability champions at ­different career stages (medical students, trainees, qualified GPs, those nearing retirement) would enable this.

## Investment in premises and capacity building

It was widely recognized that NHS estates will need to be retrofitted and rebuilt, and waste streams will need optimization to effect change at local board level and nationwide. While a degree of independence in estate management exists amongst GP teams, participants highlighted a significant need for sustainability assessments to be rolled out consistently and collectively, and for skilled teams to examine building energy supplies, insulation and waste management streams as well as carry out detailed data collection. With current unmanageable workloads in primary care, it is unrealistic to expect GP teams to do this work out of goodwill.

There is a need for dedicated funding to support sustainable general practice. At present, NHS Health Boards have sustainability governance groups with very little input from general practice. Initiatives such as NHS England’s Healthier Futures Action Fund and RCGP’s Net Zero Primary Care Development programme^24^ are welcomed, but further funding for Primary Care Sustainability Leads would help support early adopters and networks of good practice and allow GP input and governance for sustainable change.

## Next steps

The Scottish Deep End Group supports ongoing collaboration between key stakeholders to deliver more sustainable general practice. The linked issues of climate change and health inequalities require proportionate resourcing. Indeed, many existing Deep End projects—practice-attached Community Links Workers Programme^25^ and welfare advisors,^26^ Govan SHIP^27^ and the Pioneer Scheme^28^—all aim to strengthen communities, address unmet patient needs, and free up GP time for more targeted interventions. These initiatives allow GP teams to build stronger ties with local third sector organizations and support community health and wellbeing through nonpharmacological means. Commitments made for NHS Scotland to be “net-zero” by 2040^29^ will only be achieved if general practice is on board, but additional resource is required where needs are greatest, or health inequalities will inevitably widen.

## Data Availability

Not applicable.
